# Characterisation of Nuclear Architectural Alterations during *In Vitro* Differentiation of Human Stem Cells of Myogenic Origin

**DOI:** 10.1371/journal.pone.0073231

**Published:** 2013-09-03

**Authors:** Natalia Rozwadowska, Tomasz Kolanowski, Ewa Wiland, Marcin Siatkowski, Piotr Pawlak, Agnieszka Malcher, Tomasz Mietkiewski, Marta Olszewska, Maciej Kurpisz

**Affiliations:** 1 Department of Reproductive Biology and Stem Cells, Institute of Human Genetics, Polish Academy of Sciences, Poznan, Poland; 2 Institute for Biostatistics and Informatics in Medicine and Ageing Research, Medical Faculty, University of Rostock, Rostock, Germany; 3 Faculty of Animal Breeding Biology, Poznan University of Life Sciences, Poznan, Poland; 4 Traumatology and Orthopaedics Department, District Hospital of Wielkopolska, Poznan, Poland; 5 German Center for Neurodegenerative Diseases, Rostock, Germany; CNRS UMR7275, France

## Abstract

Cell differentiation is based on a synchronised orchestra of complex pathways of intrinsic and extrinsic signals that manifest in the induced expression of specific transcription factors and pivotal genes within the nucleus. One cannot ignore the epigenetic status of differentiating cells, comprising not only histones and DNA modifications but also the spatial and temporal intranuclear chromatin organisation, which is an important regulator of nuclear processes. In the present study, we investigated the nuclear architecture of human primary myoblasts and myocytes in an *in vitro* culture, with reference to global changes in genomic expression. Repositioning of the chromosomal centromeres, along with alterations in the nuclear shape and volume, was observed as a consequence of myotube formation. Moreover, the microarray data showed that during *in vitro* myogenesis cells tend to silence rather than induce gene expression. The creation of a chromosome map marked with gene expression changes that were at least 2-fold confirmed the observation. Additionally, almost all of the chromosomal centromeres in the differentiated cells preferentially localised near the nuclear periphery when compared to the undifferentiated cells. The exceptions were chromosomes 7 and 11, in which we were unable to confirm the centromere repositioning. In our opinion, this is the first reported observation of the movement of chromosomal centromeres along differentiating myogenic cells. Based on these data we can conclude that the myogenic differentiation with global gene expression changes is accompanied by the spatial repositioning of chromosomes and chromatin remodelling, which are important processes that regulate cell differentiation.

## Introduction

Skeletal muscle tissue, due to its function and exposure to potential trauma, contains naturally occurring reservoir cells called satellite cells. Being located at the basal lamina of muscle fibre, these cells constitute the source for muscle growth or regeneration following injury or exercise. Activated cells (myoblasts) proliferate, migrate and fuse with each other or with existing myocytes, giving rise to newly formed muscle fibres [1].

Myoblasts are a very promising tool for regenerative cell therapy, mainly due to the ease of isolation, the relatively high proliferation potential observed *in vitro* and the ability to colonise and interact within the target tissues [2]. Treatment of skeletal muscle disorders such as DMD (Duchenne Muscular Dystrophy) is the priority, but the myoblasts could also prove to be promising therapeutic agents in ischaemic heart disease by improving cardiac function or ameliorating defects in sphincter function in both the digestive and urinary systems [3]–[4].

The biology of myoblasts as activated stem cells involves several phases of activity through which the process of cell fusion results in differentiated muscle fibres. Stem cell differentiation is a very complicated process comprising changes in the expression profile, cell shape or displacement as well as changes in its epigenetic status, which is a crucial factor determining stem cell fate [5].

Genetically, the synchronised orchestra of myogenic transcription factors such as MyoD, Myf6, Myf5, Myogenin and their target genes are responsible for proliferation, cell cycle and cell fusion during terminal differentiation [6]. Moreover, apart from changes in gene expression, the epigenetic status of these cells seems to influence the differentiation process [7]. A genome-wide epigenetic study revealed that during the differentiation of C2C12 cells, dynamic changes are reflected in histone modification [8]. The epigenetic status seems to be tightly correlated with chromatin dynamics and may influence its plasticity, which can be estimated by observing the mobility of histone proteins [9].

Recent findings support the concept of chromosome territories, the functional compartment of the nucleus and nuclear architecture as the factors defining global gene transcription levels and cell fate [10]. The nuclear positioning of chromosomes seems to be a non-random event and correlates with specific gene expression, which corroborates the observations regarding the distinct architectural organisation of the nuclei in various cell types [11]. Such nuclear architecture may indicate a relationship between the gene expression profile and chromosome positioning in the interphase nuclei of skeletal muscle stem cells.

In this study, we used primary human myoblasts to study the position of selected chromosome centromeres (1, 3, 7, 11, 12, 17, X) in a three-dimensional nuclear structure during their *in vitro* differentiation into myocytes. We evaluated the profile of myogenic gene expression in both myoblasts and differentiated cells and simultaneously immunostained the cells for desmin, myosin heavy chain and alpha-actinin. Our goal was to clarify whether the myogenic differentiation may alter the architecture of the nucleus and if observed changes in the expression profile may influence the movement of chromosomal centromeres.

## Materials and Methods

### Cell Isolation, *in Vitro* Culture and Differentiation

Human myoblasts were isolated from muscle tissue fragments collected during ACL (Anterior Cruciate Ligament) orthopaedic surgery. These procedures were conducted in accordance with the permission granted by the Local Bioethical Committee and the Medical University of Poznan, and written consent was obtained from the study participants.

Cells were isolated as previously described. Briefly, the muscle tissue was minced and treated with a solution of 0.02% collagenase in HBSS (Hank’s Balanced Salt Solution, Lonza, Basel, Switzerland) and subsequently filtered through a 80 µm mesh. The resulting cells were washed, centrifuged and plated in a gelatin coated T-flask. Gelatin coated surface was applied only during the first week of myoblast culture and after establishing primary myoblast cell line *in vitro* culture was continued on non-coated surface. The myoblast medium was based on DMEM (Dulbecco Modified Eagle Medium) (with 4.5 g/l glucose, Lonza, Basel, Switzerland) supplemented with 20% FBS (Fetal Bovine Serum; Lonza, Basel, Switzerland), 1% Ultraglutamine (Lonza, Basel, Switzerland) and antibiotics (Lonza, Basel, Switzerland). Various concentrations of bFGF (basic Fibroblast Growth Factor; Sigma-Aldrich, St. Louis, USA) were added to the stimulated myoblasts to sustain *in vitro* proliferation. The cells were passaged at 75% confluence to avoid spontaneous myoblast fusion. The medium was treated with 2% horse serum (Sigma-Aldrich, St. Louis, USA) to induce myocyte differentiation. All *in vitro* cultures were conducted at 37°C in 5% CO_2_ atmosphere.

On the contrary to plastic culture dishes used in standard *in vitro* culture the glass surface did not support myoblast fusion and/or myocytes formation. We tested therefore several coating agents including poly-L-lysine, collagen, gelatin and Matrigel (BD, Franklin Lakes, USA) (data nota shown). Only the Matrigel® surface provided a suitable milieu for cell adherence and differentiation. So, thin layer of Matrigel was applied according to manufacturer’s protocol in all FISH and immunofluorescence experiments.

To confirm the myogenic origin of cultured cells, CD56 antigen expression was evaluated in the myoblast population under study, using the PE-conjugated CD56 antibody (Beckmann Coulter, Brea, USA) according to the manufacturer’s protocol. We analysed 10^4^ cells on the Cell Lab Quanta cytometer (Beckmann Coulter, Brea, USA).

To evaluate the myogenic potential of cell populations obtained we evaluated the fusion index (F_i_) after 7 d myoblasts differentiation both on cell culture dishes and on coverslips.

F_i_ has been defined as a ratio of nuclei number present in differentiated myotubes (N_d_) and nuclei in undifferentiated cells (N_ud_).

FI = N_d_/N_ud._To estimate a fusion index, the nuclei of differentiated cell populations were fixed in methanol:acetic acid (3∶1) for 15 min, washed in 1×PBS and stained by 10% of Giemsa solution (Merck, Germany) for 30 min.

### IF- Immunofluorescence

The cells were seeded on coverslips and after 24 hrs (myoblasts) or 7 days (myocytes), cells were fixed with 4% PFA (paraformaldehyde; Sigma-Aldrich, St. Louis, USA) during a 15 min incubation 4°C. The cell membranes were permeabilised with Triton X-100 (15 min, 0.1%, room temperature - RT) (Sigma-Aldrich, St. Louis, USA ) and the unspecific antigens were blocked for 30 min by incubating with goat serum (10% in PBS – Phosphate Buffered Saline; Sigma-Aldrich, St. Louis, USA). The appropriate dilutions of primary antibodies were prepared in a blocking solution (anti-desmin 1∶200 (Sigma-Aldrich, St. Louis, USA); anti-myosin heavy chain 1∶400 (Sigma-Aldrich, St. Louis, USA); anti-alpha-actinin 1∶500 (Sigma-Aldrich, St. Louis, USA)), and incubated overnight with the specimens at 4°C. The coverslips were then washed three times with PBS and were incubated with a secondary anti-mouse IgG antibody conjugated with FITC 1∶2000 (Abcam, Cambridge, UK). A DAPI anti-fade solution was used as a counterstain to visualise the nucleus.

### Fluorescent in Situ Hybridisation – Fish

Cells were plated on coverslips. Myoblasts were kept on slides for 24 hrs before fixation. However, to obtain myocytes, the cells were incubated in growth medium until an appropriate confluence was achieved (i.e., 90%). Horse serum was added to the culture medium to induce the myotubes formation. Finally, the slides were left until the differentiated myocytes were formed (one week).

Cells were fixed with 4% PFA in PBS and permeabilised with a 0.5% Triton X-100 solution (Sigma). Cells were then incubated in a 20% glycerol solution and repeatedly frozen in LN_2_ (liquid nitrogen). Slides were treated with 0.1 N HCl to facilitate probe penetration and kept in 50% formamide/2x SSC (Saline-Sodium Citrate) at 4°C for the next week. Finally, the cells were washed in 2x SSC and dehydrated in ethanol (70%; 85%; 96%). Slides were then left at RT (room temperature) to dry and stored at −20°C.

The fluorescent in situ hybridisation for centromeres on chromosomes 1, 3, 7, 11, 12, 17 and X was conducted according to the manufacturer’s protocol. Briefly, the directly labelled human alpha-satellite probes (CytoCell, Cambridge, UK) were pre-warmed at 37°C, together with the studied specimen, and subsequently denatured. Following overnight hybridisation in a humidified, lightproof chamber at 37°C, the slides were washed twice (at 72°C and at RT, in 2x SSC buffer). DAPI solution was used as a counterstain and the slides were evaluated under a confocal microscope.

### Bioinformatic and Statistical Analysis of 3D FISH

**Figure 1:**
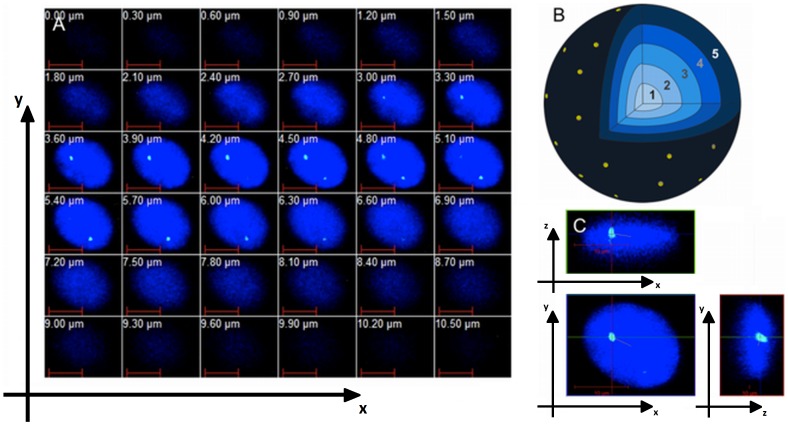
Scheme of 3D analysis of interphase nuclei. A – stack of pictures collected in the Z-axis; B – model of 3D reconstruction of the nucleus with a schematic view of the co-centric shells; C – evaluation of centromere position in the 3D nucleus environment.

The stacks of pictures for 3D FISH analysis (50+/−8 cell nuclei/cell population) were derived using an Axiovert 200 fluorescent confocal microscope (Zeiss, Jena, Germany), analysed by LSM Image Browser (Zeiss, Jena, Germany) and subsequently by NEMO [12]. For each cell, volume and flattening were measured. The relative distance of the centromere hybridisation signal was calculated from the nuclear centre, based on the division of the nuclear diameter on equal sections (Figure 1). Volume and flattening samples for both, myoblasts and myotubes, did not undergo a normal distribution (Shopiro-Wilk test for normality, p-value<0.001 for all four samples), therefore, we decided to use a non-parametrical analysis. Statistical significance of a morphological analysis of the nuclei volume and the flattening was calculated by Mann-Whitney U test. The nucleus of each cell was divided into five co-centric shells of the same volume. Then, centromeres signals were assigned to appropriate shells, creating a signal distribution in cell nuclei. The centromere signal distribution was then analyzed with the chi-square test.

### Microarray Assay

RNA was obtained from *in vitro* cultured myoblasts and myocytes using the TriReagent (Sigma-Aldrich, St. Louis, USA) according to the manufacturer’s protocol. Evaluation of RNA integrity and quantity was conducted using the BioAnalyser (Agilent, Santa Clara, USA) and the NanoDrop spectrophotometer (Nanodrop, Wilmington, USA). The samples were prepared in triplicate.

The single stranded DNA was generated using the Ambion WT Expression Kit (Ambion, Austin, USA), followed by fragmentation and labelling of cDNA using the Affymetrix GeneChip WT Terminal Labelling Kit (Affymetrix, Santa Clara, CA). Array hybridisation, washing and scanning were conducted according to manufacturer’s protocol using high-density arrays GeneChip Human Gene 1.0 ST (Affymetrix, Santa Clara, CA). After washing, microarrays were scanned using the GeneChip Scanner 3000 (Affymetrix, Santa Clara, CA). The data shown in this publication have been deposited in NCBI’s Gene Expression Omnibus and are accessible through GEO Series accession number GSE45819 (http://www.ncbi.nlm.nih.gov/geo/query/acc.cgi?acc=GSE45819).

### Data Analysis

The CEL files obtained were analysed with the Expression Console software (Affymetrix, Santa Clara, CA) by using Robust Multi-chip Analysis (RMA) to correct the background. Quantile normalisation was performed and the results obtained were normalised while the adjusted set of data was expressed on a logarithmic scale. For subsequent analysis, Subio software (Subio, Japan) was used. Differential expression analysis was conducted for transcripts showing at least a 2-fold difference between groups and statistical significance was determined by the Benjamini-Hochberg algorithm for multiple testing to adjust the p-value defined by the t-test (p<0.01).

## Results

### *In Vitro* Culture of Myoblasts

**Figure 2:**
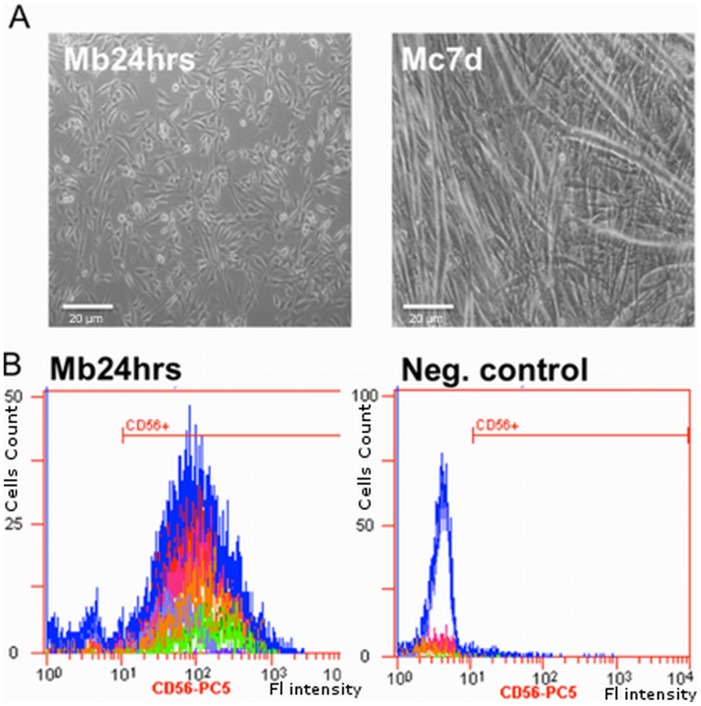
Human myoblasts in *in vitro* culture. A – flow cytometry analysis of CD56 expression on human myoblasts. Mean percent of cells positive for the CD56 marker: 85.0+/−5.0%. Panel B *- in vitro* culture of myoblasts and myocytes (Mb24 hrs – proliferating myoblasts; Mc7d- fused myocytes (myotubes). magnification-100x).

Isolated myoblasts were tested for their differentiation potential and characterised as CD56+positive when expression levels between 80%–90% were observed for the cell populations under study (Figure 2A). The morphology of cells that had differentiated into myocytes changed rapidly, from fibroblast-like mononuclear cells to strongly elongated fused multinucleated myotubes (Figure 2B). The calculated fusion index showed efficient *in vitro* myocytes formation (F_i_ = 4.5±0.25). (Figure S1).

Several attempts were made to obtain well-developed myotubes on non-plastic (glass) surfaces by coating coverslips and slides with gelatine (0.1% and 1%), collagen or poly-L-lysine solutions. Moreover, we tested different culture chambers (Sigma-Aldrich, St. Louis, USA) and *in vitro* cultures were carried out on plastic coverslips. None of these efforts fulfilled our expectations (data not shown). Our experiments clearly showed that the use of a thin layer of Matrigel® (BD, Franklin Lakes, USA) created the optimal conditions for myoblast differentiation on glass coverslips (Figure 2B). Therefore, this protocol of cell differentiation was used for all the subsequent experiments.

### IF- Immunofluorescence

**Figure 3:**
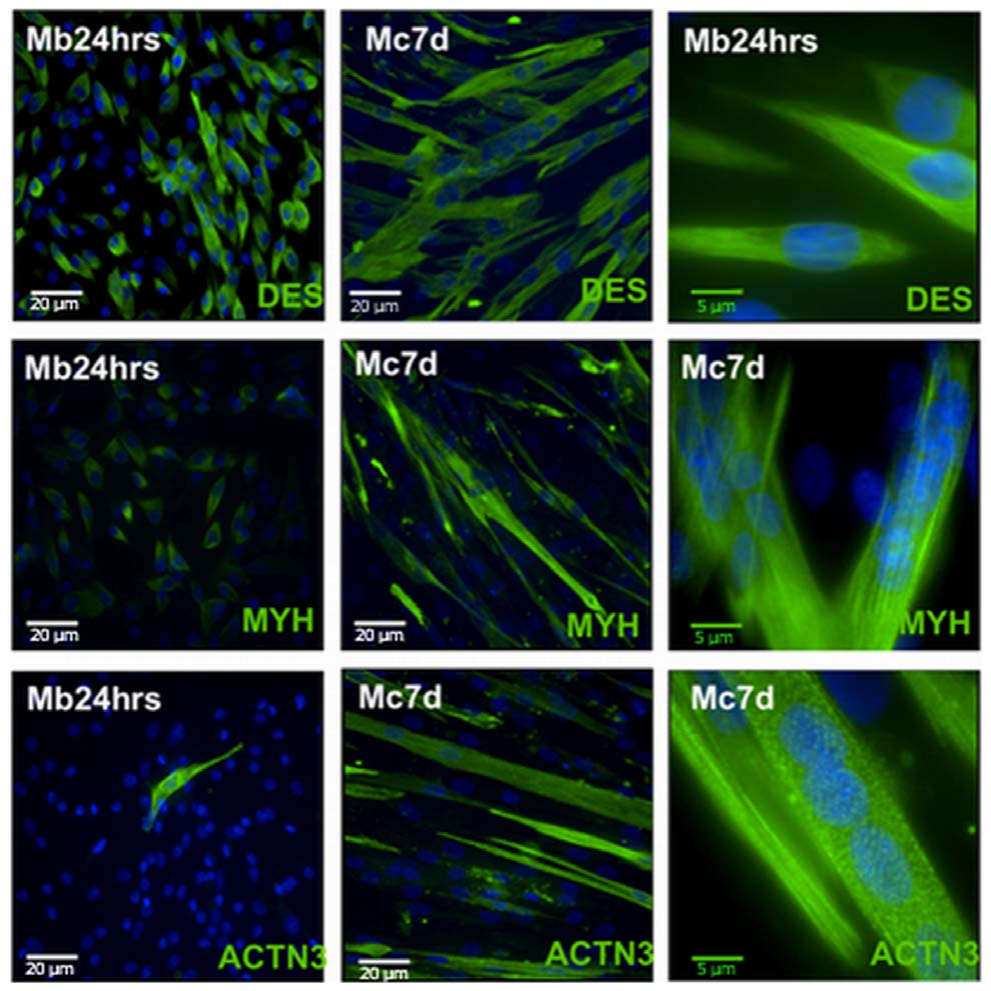
Immunofluorescence analysis of desmin (DES), myosin heavy chain (MYH) and alpha-actinin (ACTN) in myoblasts (Mb24 hrs) and myocytes after 7 days of differentiation (Mc7d). (Magnification in the left and middle columns-200x; in the right column 630x).

We evaluated the expression of myosin heavy chain (MYH), desmin (DES) and actinin (ACTN) in different populations of myoblasts and myocytes (Figure 3). The human proliferating myoblasts showed a high expression of desmin filaments, confirming their myogenic origin, and this expression was further maintained following cell differentiation. Some of the myoblasts also expressed MYH, although the signal intensity was lower compared to myocytes, most likely due to a more diffused distribution. On the contrary, ACTN was present only in the myocytes. Surprisingly, we observed actinin-positive myoblasts, which were most likely correlated with changes in the cell shape and spontaneous differentiation. The fraction of actinin-positive cells within the myoblast cell population was negligible.

### FISH Analysis

**Figure 4:**
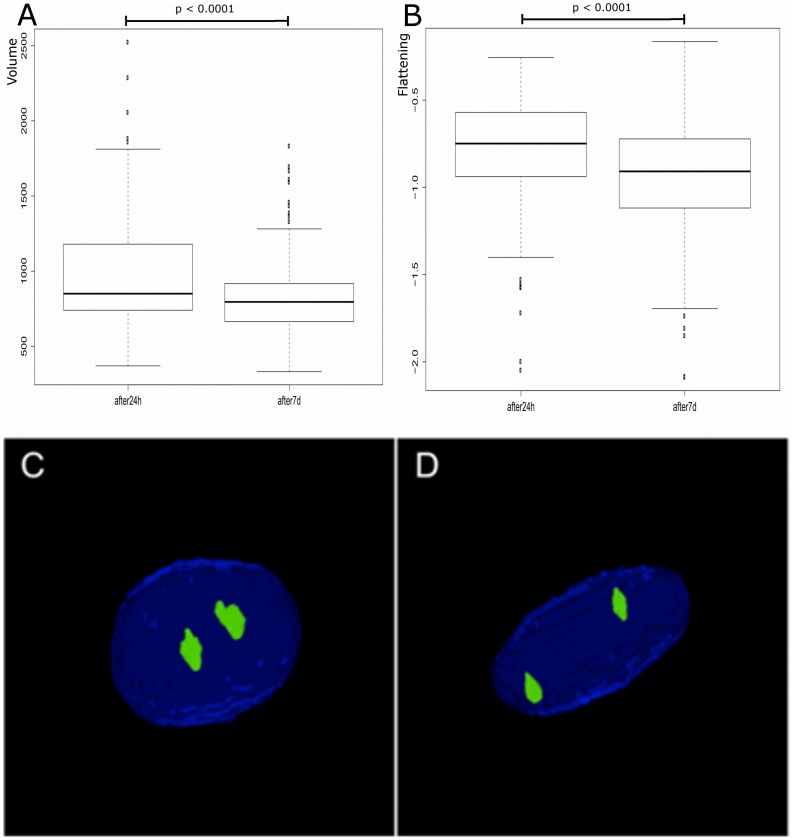
Morphological comparison of myoblast and myotube nuclei. Nuclei of differentiated myotubes have been characterised by a smaller volume (A) and increased flattening (B) compared to proliferating myoblasts. In both cases, differences were statistically significant (p<0.00001). C – 3D projection of myoblast D – myotube nucleus with a signal detected for chromosome centromeres.

In the first phase of the analysis, two types of nuclei, myoblasts (Mb24 h, n = 316) and myocytes (Mc7d, n = 379), were investigated to compare the morphological parameters of their volumes and flattening. As a result, the nuclei of myotubes appeared to be smaller and more flattened compared to the nuclei of myoblasts (p<0.0001), as shown in Figure 4.

**Figure 5:**
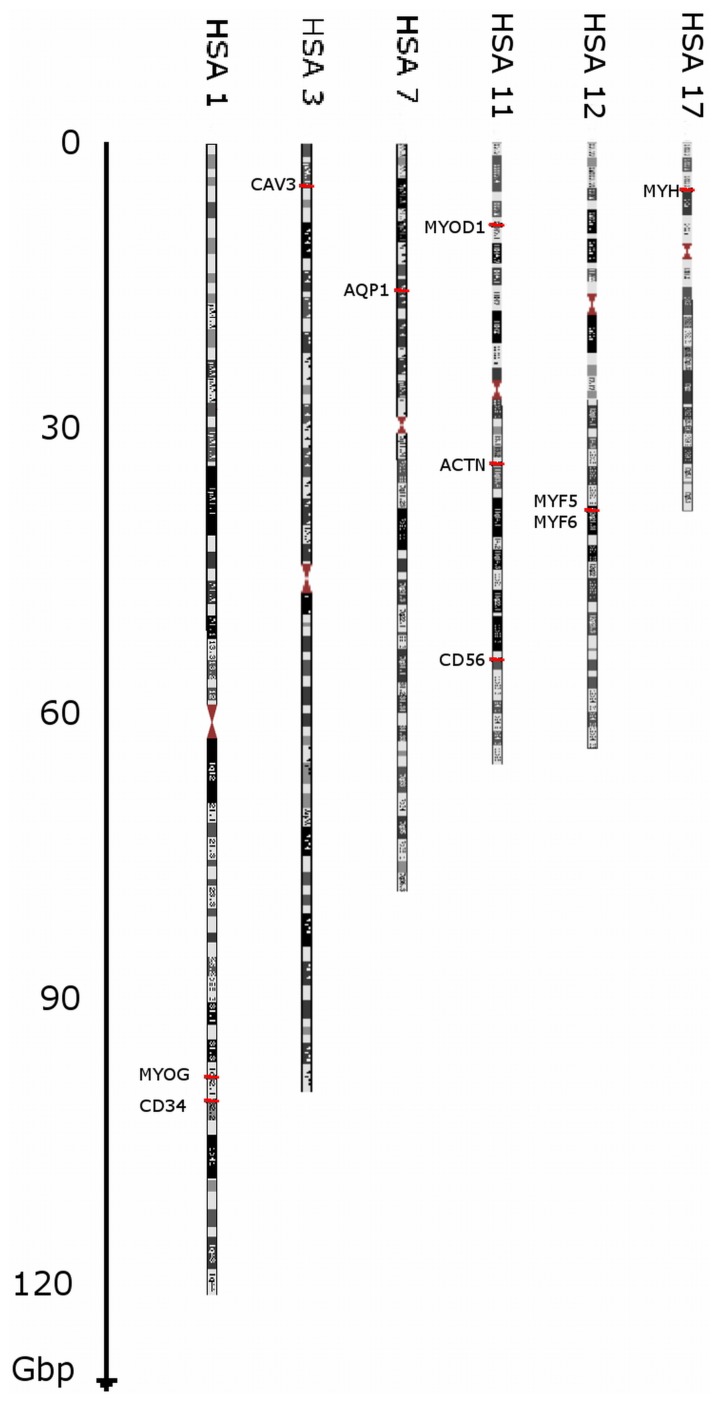
Cytogenetic maps of chosen chromosomes along with genes (loci) of interest marked. HSA1(MYOG, CD34), HSA3 (CAV3), HSA7 (AQP1); HSA11 (CD56, MYOD, ACTN3); HSA12 (MYF5, MYF6); HSA17 (MYH); HSAX.

**Figure 6:**
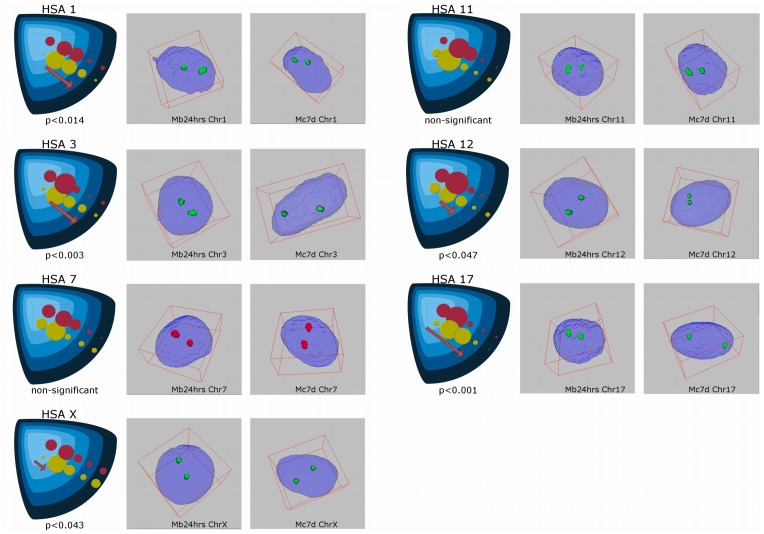
Comparison of the changes in chromosome centromere distribution, before (myoblasts Mb24 hrs − red spots) and after (myocytes Mc7d − yellow spots) cell differentiation. When analysing chromosomes 7 and 11, the observed changes did not reach statistical significance (p>0.05). In other cases, the observed changes in centromere localisation were directed towards the nuclear periphery (chromosomes 1, 3, 12, 17, X).

For 3D FISH evaluation (Figure 5), chromosomes were chosen according to the position of genes known to play a crucial role in the maintenance of myogenic characteristics of cells. The nuclear position of the centromeres on chromosomes 1, 3, 7, 11, 12, 17 and X were investigated at two time points: during active proliferation of myoblasts in culture and in terminally differentiated, non-dividing multinuclear myotubes. Clearly, changes in the nuclear localisation of the centromeres on five chromosomes (1, 3, 12, 17, X) were observed (p<0.05), whereas the centromeres of chromosomes 7 and 11 seemed to remain stable (>0.05). We could distinguish three schemes of centromere re-localisation; the first of these applied to the centromeres of chromosomes 1, 3 and 17, with a well–defined movement towards the nuclear border, whereas the centromeres of chromosomes 12 and X were preferentially found in the intermediate shells of the nuclei, which defined the second type of re-localisation. The last group consisted of the centromeres of chromosomes 7 and 11, which were rather stable in their nuclear position. The apparent changes in the location of the centromere of chromosome 1 were noticed in the first shell, in which 19.77% of the signal in myoblasts was observed, whereas only 4.55% of the signal was observed in myocytes. The same phenomena were observed in the case of chromosome 3; almost 26% of the centromeres were located in the inner shell in the undifferentiated cell state. However, after myocyte formation, only 7.89% of the centromeres were found in the centre of the nuclei. The arrangement of the centromeres of chromosomes 7 and 11 did not change during myogenesis. The outermost shell seemed to be rather unoccupied (from 1.89% to 9.82% centromeres of the investigated chromosomes), with the exception of the chromosome X centromere, in which 14.58% (myoblasts) and 19.15% (myocytes) of the centromeric signals were detected. Interestingly, 10% of the centromeres of chromosome 12 were found in the outer shell of myocyte nuclei, but no centromeres were localized in myoblasts. The centromere of chromosome X also seemed to have moved towards the nuclear lamina during myogenesis; 23.96% of the centromere signals were located in the inner shell during myogenesis and only 7.45% were found in the inner shell after differentiation. The data have been summarised in Table 1 and illustrated in Figure 6.

**Table 1:** Comparison of chromosome centromere distribution in co-centric nuclear shells in myoblasts (24 hrs after cell plating) and in differentiated myocytes (after 7 days in a medium containing 2% horse serum).

Chromosome	Time	First Shell	Second	Third	Fourth	Fifth
**HSA1**	After 24 hrs	19.77%	31.40%	30.23%	9.30%	9.30%
	After 7 days	4.55%	39.77%	31.82%	18.18%	5.68%
**HSA3**	After 24 hrs	25.68%	44.59%	14.86%	6.76%	8.11%
	After 7 days	7.89%	41.23%	25.44%	17.54%	7.89%
**HSA7**	After 24 hrs	28.26%	34.78%	23.91%	9.78%	3.26%
	After 7 days	23.44%	41.41%	12.50%	11.72%	10.94%
**HSA11**	After 24 hrs	15.79%	42.98%	29.82%	7.89%	3.51%
	After 7 days	18.10%	49.14%	15.52%	8.62%	8.62%
**HSA12**	After 24 hrs	29.41%	48.53%	14.71%	7.35%	0.00%
	After 7 days	23.21%	34.82%	23.21%	8.93%	9.82%
**HSA17**	After 24 hrs	29.41%	45.10%	12.75%	7.84%	4.90%
	After 7 days	12.26%	40.57%	34.91%	10.38%	1.89%
**HSAX**	After 24 hrs	23.96%	32.29%	17.71%	11.46%	14.58%
	After 7 days	7.45%	38.30%	22.34%	12.77%	19.15%

From the nuclear interior to the periphery, 5 shells were identified.

### Microarray Assay

**Figure 7:**
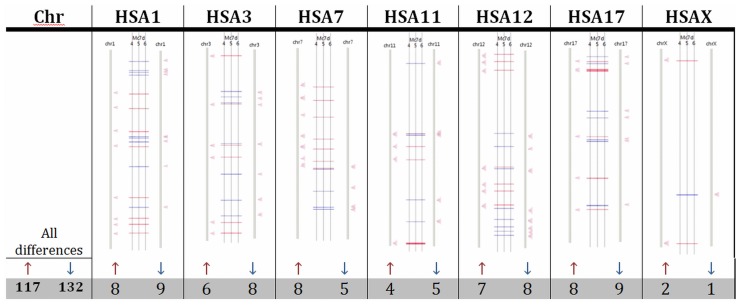
Schematic view of expression changes (2-fold) obtained by microarray analysis. Global expression found in all chromosomes showed an increase in gene silencing during in vitro myogenesis (132 transcripts down-regulated vs. 117 transcripts up-regulated). The up-regulated transcripts are in red and the down-regulated transcripts are in blue. The chromosomes subjected to 3D FISH evaluation are shown in schematic ideograms.

To study changes in the global transcriptome and to correlate the observed centromere repositioning with genes being switched-on/off, we used microarray gene expression analysis. Among the investigated genes, there were 249 transcripts with a 2-fold change in expression in the differentiated myogenic cells compared to the myoblasts. Up-regulation of expression was observed for 117 transcripts, while 132 were silenced (Figure S2 and Table S2). Subsequently, the marked genes were put on chromosome ideograms, and the evaluation was conducted only on chromosomes investigated in the 3D FISH experiments. The observed pattern of up- and down-regulated genes was surprisingly uniform. The chromosomes 1, 3, 11, 12, 17 and X contained an almost equal number of up-regulated genes (from 8 to 6), and one more gene was found to be consistently silenced (from 9 to 7). Chromosome 7 did not follow the rules in which the coding sequences for 8 active and 5 silenced genes were marked. Figure 7 (Table S1) summarises the data that were obtained.

## Discussion

The skeletal muscles are capable of growing and regenerating as a result of tissue commitment processes followed by stem cells that reside between muscle fibres. These myogenic populations are composed of cells with a distinct expression profile, different origin and miscellaneous regeneration potential. In this study, we investigated the cell population obtained from an adult human skeletal muscle. The same protocol was applied for myoblast preparation in the 1^st^ phase of the clinical trial regarding stem cell therapy for an infarcted myocardium. The protocol allowed for a myoblast population with a high expression of CD56, the myogenic marker widely used to identify cells with a high myogenic potential [13]. In fact, the purpose of this experiment was not only to investigate the correlation between centromere position and myoblast differentiation but also to obtain a more detailed characterisation of cells that were used for infarcted myocardium therapy.

As the process of *in vitro* myoblast differentiation on standard cell culture surfaces did not provoke any problems, we did not expect that to obtain proper myocyte specimens on coverslips, we would have to test several surface coatings (gelatin, collagen, poly-L-lysine). We observed that Matrigel® was the only surface coating agent that stimulated myotube formation. It was previously shown that Matrigel® created an optimal environment for the *in vitro* 2D and 3D culture of muscle cells, and it also stimulated the myogenic potential of muscle progenitor cells and stabilised myogenic gene expression (MyoD, MyoG) [14].

To ensure the quality and quantity of ongoing myogenesis on coverslips, we evaluated the expression of several important markers: DES, MYH and ACTN. Desmin is known to be one of the key markers of muscle commitment [15]. The starting myoblast population expressed desmin and MYH, but as shown by other investigators, we found that MYH expression is strongly up-regulated after myocyte formation and desmin can still be found in myotubes regardless of the differentiation process [16]. The evidence for proper organisation of myotubes was provided by the presence of cytoplasmic ACTN filaments - a major structural component of the sarcomeric Z line in mammalian skeletal muscle [17].

There is increasing evidence showing that besides the transcription factor network governing cell fate, the genome undergoes epigenetic changes during cell differentiation, which too are crucial in determining cell fate. These changes are not only based on DNA methylation and histones modifications but also on heterochromatin formation, and the spatial and temporal positioning of chromosome in the nuclear space. Our confocal, 3D analysis of myoblast nuclei vs. myocyte nuclei showed that during myogenic differentiation, interphase nuclei had smaller volumes and were more flattened in shape. These results are consistent with reports in a mouse model of myogenesis that used C2C12 cells. A 2D analysis showed that the nuclei of mouse myoblasts changed upon differentiation, as observed by a reduction in the nuclear area and a change in the overall nuclear morphology [9]. Indeed, the nuclei of pluripotent cells such as ES cells are relatively large and almost devoid of heterochromatin, thus defining their extreme plasticity and an “open” chromatin state. During differentiation, the compact heterochromatin concentrates in distinct foci [18]. Similarly, as myogenesis advances, the cellular nuclei become more compact most likely due to the accumulation of rigid heterochromatin [19].

The chromosome and centromere position are considered to be important for nuclear organisation and creating another important factor in an epigenetic landscape of the cells. In our view, this is the first report in which centromere movement has been documented along the differentiation of stem cells of myogenic origin. There have been several examples showing that chromosomes, during *in vitro* cell differentiation, change their positions in distinct cell types. Our evaluation of the chromosome centromere topology during *in vitro* myogenesis clearly showed that the nucleus of differentiating myoblasts is a very dynamic structure. We demonstrated that the positions of the centromeres of chromosomes 1, 3, 12, 17, X were changing and the centromeres were most often found near the nuclear periphery rather than near the centre of the nuclei after differentiation. Our previous 2D study showed a similar pattern of chromosome X relocation [20]. We did not distinguish active and inactive (Xa and Xi) chromosome position. It was shown by others that during differentiation of hES cells (with retinoid acid) both Xa and Xi changed their position toward nuclear periphery with more pronounced Xi relocation. [21] The centromeres of chromosomes 7 and 11 did not alter their intranuclear layout during an *in vitro* myogenesis. Chaly and Munro first provided the chromosome topology observations during myogenesis [22], wherein they described the centromeric pattern in the interphase nuclei. Our findings support their data, and in our hands, five of seven investigated chromosomes preferentially localised towards the nuclear border. In our study, we tracked the chromosomal centromeres instead of chromosome territory (CT). The preferential localisation of chromosomes due to their size or number of genes was investigated in many cell types. An attempt to establish one general chromosomal pattern failed, but some of our findings support the concept that gene-rich chromosomes (1, 16, 17, 19 and 22) are found in the nuclear interior in contrast to gene-poor chromosomes (2, 4, 13, 18) that mostly reside close to the nuclear border [23]. It was previously shown that the nuclear chromosome position and chromosome size are correlated; the larger chromosomes are predominantly peripherally located in the nucleus [24]. We could apply these findings to our model and compare the position of the large chromosome 1 with that of the smaller chromosome 17. The chromosome 1 in myoblasts was found in the middle shells of the nucleus, with a marked tendency to move towards the nuclear periphery after differentiation. Chromosome 17 during myogenesis also demonstrated a similar tendency, but 74% of the centromeres localised in the first two inner shells, and they could be found in the intermediate compartment of the nucleus after differentiation.

A number of studies support the importance of chromosome position in the interphase nuclei with relation to gene expression [25] [26]. The correlations between the expression profile and modification of epigenetic status of chromatin are often mentioned with reference to nuclear architecture. It is believed that the nuclear periphery is transcriptionally silenced, whereas the transcription machinery seems more active in the nuclear interior [27]. However, there are several exceptions to this model, and many authors have shown that some genes are expressed near the nuclear periphery, while others located in the interior of the nucleus are not expressed [28]. The map of the Human Transcriptome revealed a clustering of highly expressed genes (ridges) and weakly expressed genes (antiridges) [29]. The three-dimensional structure of this domain showed that ridges are less condensed and localise in the nuclear interior, while the antiridges are compact, regular in shape and frequently associate with the nuclear periphery [30]. Contradictory data were obtained regarding domain-driven tissue-specificity. The comparison of ridges/antiridges regions in different cell types showed that these genomic domains are independent of expression profile or differentiation state, although there is some evidence that tissue-specific genes are present both in silent and expression active domains [31]. Evaluation of the global myogenic cell transcriptome by microarray confirmed the correlation between cell differentiation and the downregulation of gene expression. The same observation was made during granulopoiesis, and it was accompanied by non-random tissue-specific chromatin condensation [32]. We created a chromosome map marking genes with at least a 2-fold change in expression on chromosomes evaluated earlier in 3D FISH experiments. The up- and down-regulated genes were localised in all the investigated chromosomes. An important question was raised with regards to the relationship between chromosome re-positioning and gene activation or silencing. Based on 3D FISH, the chromosomes that were studied, with the exception of HSA7 and HSA11, showed a tendency to localise in the outer shell of the nucleus after differentiation. There was a balance between the up- and down-regulated genes found on particular chromosomes (silenced vs. up-regulated genes e.g., 7 vs. 8), which have a tendency to reposition, but this was not observed with chromosome 7. Chromosome 7 did not change the localisation pattern during myogenesis and we could speculate whether the advantage of gene up-regulation over gene silencing (8 vs. 5) resulted in this effect. On the other hand, we did not notice any changes in the localisation of HSA11 centromeres, although the chromosome contained almost equal number of genes with over a 2-fold change in expression. At first glance, this phenomenon seems inconsistent with our findings, but one must acknowledge that epigenetic factors that influence gene expression, together with spatial and temporal nuclear organisation during cell differentiation, most likely create the unique network. Further evaluations of the differences in histones modification, the presence of heterochromatin foci or quantitative analysis of non-histone proteins (e.g., transcriptional machinery proteins) could potentially clarify the observed discrepancy between the intranuclear behaviour of chromosomes. Emerging evidence of centromere (regulatory) positioning factors was obtained from our recent analysis of the microarray data (data not shown) demonstrating intense transcriptional activity in the pericentromeric region of chromosome 11; this was strikingly different from the remaining chromosomes that were analysed. This could possibly explain why chromosome 11 exhibited a different stability and a distinct pattern from other migrating chromosomes studied. Widespread regions of active chromatin could potentially influence the centromere position within the cell nucleus, but this observation needs to be confirmed by further studies.

As mentioned earlier, myoblasts are a very promising tool in the context of regenerative therapy for many diseases. Assessment of their properties and the myogenesis process itself could provide a better understanding of the regulation of cell differentiation and could potentially improve the efficacy of stem cell therapy.

## Conclusions

Chromatin distribution and condensation is thought to be cell-type specific and relevant to spatial and temporal expression profiles. The results obtained indicate that during myogenesis, the nuclei of myoblasts are dynamic structures that despite of the nucleus compression they maintain expression of genes necessary to cell function. The nuclear architecture during interphase is an integral part of the regulation of gene expression, an important element of the epigenetic status, and it should be considered important in cell fate determination.

## Supporting Information

Figure S1 **Myocytes stained with Giemsa solution.** The calculated F_i_ show efficient myocytes formation. Arrows show the multinucleated myotubes after cells fusion.
(TIF)

Figure S2 **Summary of gene products by using gene ontology terms and extracted from the GO database and for different function subcategories.** Transcripts up-regulated (A) and down-regulated (B) during myogenesis are presented.
(TIF)

Table S1 **Annotation of differentially expressed (myoblast vs. myocytes) transcripts on HSA1, HSA3, HSA7, HSA11, HSA12, HSA17 and HSAX.**
(DOCX)

Table S2 **GEO detailed annotations.**
(DOCX)
