# Supplementary figures and images for: Characterisation of Nuclear Architectural Alterations during In Vitro Differentiation of Human Stem Cells of Myogenic Origin
Source: PLoS One. 2013 Sep 3;8(9):e73231. doi: 10.1371/journal.pone.0073231 (PMC3760906; doi:10.1371/journal.pone.0073231)

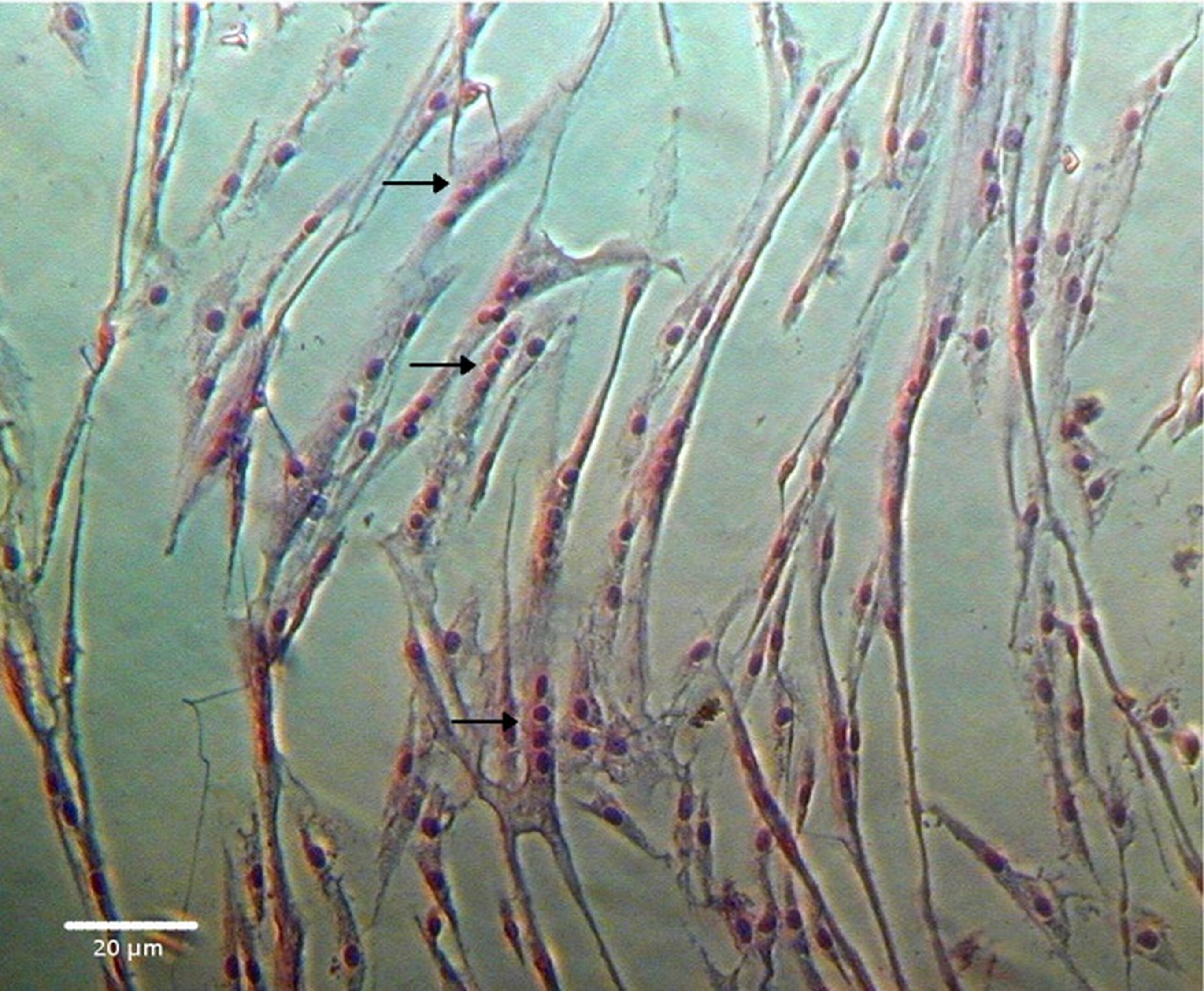

Supplement: Figure S1 — Myocytes stained with Giemsa solution. The calculated Fi show efficient myocytes formation. Arrows show the multinucleated myotubes after cells fusion. (TIF) [file pone.0073231.s001.tif]

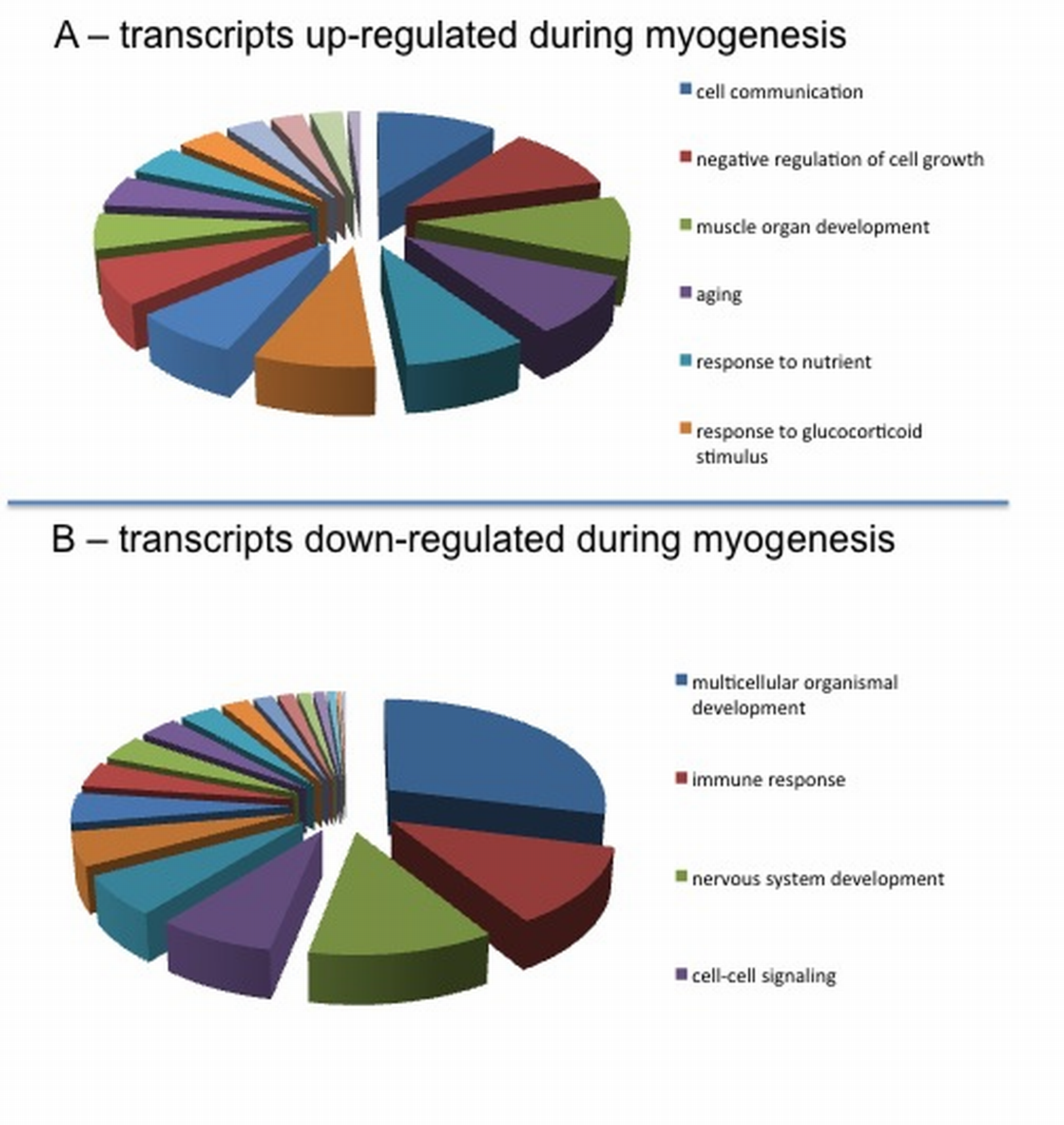

Supplement: Figure S2 — Summary of gene products by using gene ontology terms and extracted from the GO database and for different function subcategories. Transcripts up-regulated (A) and down-regulated (B) during myogenesis are presented. (TIF) [file pone.0073231.s002.tif]
