# Supplementary material for: Characterisation of Nuclear Architectural Alterations during In Vitro Differentiation of Human Stem Cells of Myogenic Origin
Source: PLoS One. 2013 Sep 3;8(9):e73231. doi: 10.1371/journal.pone.0073231 (PMC3760906; doi:10.1371/journal.pone.0073231)
Supplement: Table S1 — Annotation of differentially expressed (myoblast vs. myocytes) transcripts on HSA1, HSA3, HSA7, HSA11, HSA12, HSA17 and HSAX. (DOCX) [file pone.0073231.s003.docx]

|  | HSA1 | | HSA3 | | HSA7 | | HSA11 | | HSA12 | | HSA17 | | HSAX | |
| --- | --- | --- | --- | --- | --- | --- | --- | --- | --- | --- | --- | --- | --- | --- |
|  | **↑** up | **↓** down | **↑** up | **↓** down | **↑** up | **↓** down | **↑** up | **↓** down | **↑** up | **↓** down | **↑** up | **↓** down | **↑** up | **↓** down |
| 1 | PODN | TNFRSF1B | LMCD1 | CDCP1 | GARS | non coding | SERPING1 | NRIP3 | CCND2 | TMEM194A | XAF1 | ARRB2 | KAL1 | IL13RA2 |
| 2 | NEGR1 | ID3 | FLNB | TMEM158 | INHBA | NPTX2 | CD248 | SLC43A3 | A2M | HMGA2 | GAS7 | CD68 | BGN |  |
| 3 | VCAM1 | STMN1 | TMEM45A | MAPKAPK3 | LANCL2 | MET | GDPD5 | LPXN | MGP | MYF5 | MYH8 | SPAG5 |  |  |
| 4 | PHGDH | PTPRU | CCDC80 | ARHGEF3 | ELN | FAM40B | OPCML | MMP1 | PAWR | BTBD11 | MYH2 | EVI2A |  |  |
| 5 | FAM129A | NTNG1 | MTHFD2 | DCBLD2 | CACNA2D1 | PODXL |  | FXYD6 | EPYC | TBX3 | MYH3 | TOP2A |  |  |
| 6 | KCNH1 | GPSM2 | CLDN1 | TRH | CYP51A1 |  |  |  | NTN4 | CIT | CACNB1 | KRTAP1-1(1) |  |  |
| 7 | TGFB2 | PTPN22 |  | TIPARP | ASNS |  |  |  | ALDH1L2 | KNTC1 | ANKFN1 | KRTAP1-1(2) |  |  |
| 8 | ACTA1 | ITGA10 |  | NCEH1 | non coding |  |  |  |  | TMEM132B | ABCA6 | KRTAP1-1(3) |  |  |
| 9 |  | ASPM |  |  |  |  |  |  |  |  |  | PITPNC1 |  |  |
|  | 8 | 9 | 6 | 8 | 8 | 5 | 4 | 5 | 7 | 8 | 8 | 9 | 2 | 1 |

Table S1
