# Supplementary material for: Characterisation of Nuclear Architectural Alterations during In Vitro Differentiation of Human Stem Cells of Myogenic Origin
Source: PLoS One. 2013 Sep 3;8(9):e73231. doi: 10.1371/journal.pone.0073231 (PMC3760906; doi:10.1371/journal.pone.0073231)
Supplement: Table S2 — GEO detailed annotations. (DOCX) [file pone.0073231.s004.docx]

Table S2

| Up-regulated in Mc7d | | |
| --- | --- | --- |
| Category | **#in category** | **P-value** |
| cell communication | 98 | 0.0054343194 |
| negative regulation of cell growth | 96 | 0.0051406063 |
| muscle organ development | 95 | 1.5652547E-6 |
| aging | 87 | 0.003938879 |
| response to nutrient | 76 | 1.00063235E-5 |
| response to glucocorticoid stimulus | 75 | 9.41484E-6 |
| axon guidance | 68 | 0.0020107545 |
| wound healing | 65 | 0.0017765863 |
| notch signaling pathway | 53 | 0.001013069 |
| response to camp | 46 | 6.8542827E-4 |
| response to mechanical stimulus | 44 | 2.4662306E-5 |
| one-carbon metabolic process | 36 | 3.4876858E-4 |
| collagen fibril organization | 31 | 2.3129533E-4 |
| cellular amino acid biosynthetic process | 26 | 1.4304894E-4 |
| cellular response to hormone stimulus | 26 | 1.4304894E-4 |
| muscle filament sliping | 10 | 1.16638175E-5 |

| Down-regulated in Mc7d | | |
| --- | --- | --- |
| Category | **#in category** | **P-value** |
| multicellular organismal development | 935 | 0.02204363 |
| immune response | 399 | 9.4161616E-11 |
| nervous system development | 392 | 0.0047097607 |
| cell-cell signaling | 250 | 0.009335059 |
| anti-apoptosis | 205 | 0.04895928 |
| cell surface receptor linked signaling pathway | 170 | 0.035064645 |
| response to hypoxia | 151 | 1.4629746E-4 |
| angiogenesis | 127 | 0.0014515773 |
| positive regulation of i-kappab kinase/nf-kappab cascade | 126 | 0.020324094 |
| protein complex assembly | 107 | 0.015024517 |
| induction of apoptosis by extracellular signals | 102 | 0.013746124 |
| regulation of rho protein signal transduction | 75 | 0.007733561 |
| response to estrogen stimulus | 54 | 0.004164695 |
| bmp signaling pathway | 45 | 0.0029523182 |
| negative regulation of transcription factor activity | 35 | 0.0018389375 |
| antigen processing and presentation of peptide or polysaccharide antigen via mhc class ii | 31 | 1.6802558E-17 |
| regulation of angiogenesis | 23 | 8.393203E-4 |
| regulation of mapkkk cascade | 10 | 1.8730496E-4 |
| antigen processing and presentation of exogenous peptide antigen via mhc class ii | 10 | 1.8730496E-4 |
